# Pulmonary Function Testing in Asthmatic Children. Tests to Assess Outpatients During the Covid-19 Pandemic

**DOI:** 10.3389/fped.2020.571112

**Published:** 2020-11-17

**Authors:** Mario Barreto, Melania Evangelisti, Marilisa Montesano, Susy Martella, Maria Pia Villa

**Affiliations:** Pediatric Unit Sant'Andrea Hospital, NESMOS Department, Faculty of Medicine and Psychology, “Sapienza” University, Rome, Italy

**Keywords:** coronavirus disease (COVID-19), SARS-Cov-2, pulmonary function testing (pft), asthma, children, forced oscillation technique (FOT), interrupter resistance (Rint), exhaled nitric oxide (FeNO)

## Introduction

The worldwide Covid-19 outbreak has challenged our rules to manage pediatric patients with chronic respiratory diseases, particularly asthma. The spread of the novel coronavirus causing severe acute respiratory syndrome (SARS-Cov-2) constrained most countries to adopt drastic infection control strategies for health-care facilities (1, 2). Several Respiratory Societies have developed recommendations to protect patients and health-care staff in pulmonary function laboratories (3–6). These documents advise restriction of pulmonary function tests (PFTs) to those deemed essential, namely, spirometry and diffusion capacity measurements, in line with their clinical relevance and the epidemic phase. When testing is pertinent, infection prevention and control measures in the laboratory are required, including the disinfection of instruments, room cleaning, ventilation, and protective measures for patients and health personnel (3–6).

Notwithstanding the preceding measures, the risk of exposure to viral loads of SARS-Cov-2 following active respiratory maneuvers is not negligible. Consequently, all aerosol-generating procedures (AGPs) are discouraged, and no bronchial provocation tests are allowed.

Recommendations prevent spirometry in any child with suspected/confirmed Covid-19 (4, 6); on the other hand, the clinical picture of worsening asthma or an asthma exacerbation substantially overlaps with Covid-19 (7).

A recent consensus suggests screening patients for high-risk exposures, fever, and symptoms suggestive of COVID-19, ideally in two phases: within 3 days and upon arrival for medical visits when PFTs are due to be carried out. Alternatively, testing for SARS-CoV-2 patients within 72 h of their appointment (8). Patients reporting new symptoms upon arrival (even if previously tested negative for SARS-CoV-2) should be considered as suspected cases of COVID-19. In confirmed or suspected cases of COVID-19, resolution of symptoms and two negative tests for SARS-CoV-2 collected ≥24 h apart are required (6, 8). These patients could be rescheduled for PFTs after an interval without symptoms and fever since at least 10 days from their onset or, if asymptomatic, at least 10 days from their first positive test (8).

Execution of PFTs in patients coming from high prevalence settings [i.e., high-risk patients, require a negative pressure room (4, 8)]. The staff should wear an N95 mask, face shield, gown, and gloves; consumables must be managed within the risk area. Patients should wear a surgical mask between measures; both patients and staff should follow protocols on safe distance and hand washing. Metered dose inhalers (MDI) must replace nebulizers for assessing the bronchodilator response. Room and equipment disinfection, ventilation, and timing between patients are compulsory (4, 8).

Optional home spirometry can be useful for children needing close surveillance of pulmonary function (discussed in the last section).

## Questions on the Need for Alternative Tests

While restrictions in the pulmonary function laboratory will change as the disease prevalence changes, several questions remain: shall we continue monitoring airway patency/responsiveness (i.e., bronchodilation with beta-agonists) with spirometry as the sole measurement tool? Could we offer alternative PFTs to asthmatic children? If so, what is the rationale for considering this alternative?

Small airways dysfunction in asthma is now widely recognized (9–11). Spirometric variables such as FEV_1_ are affected by flow resistance at the large airways. This fact may explain why spirometry poorly correlates with symptoms and why only markedly decreased FEV_1_ values (<60%) predict disease exacerbations (12). On the other hand, visual inspection of flow-volume curves and assessment of bronchodilation help clinical decisions (12, 13).

Spirometry is effort dependent and requires expertise (13). However, it is just the “forced” nature of spirometry that allows enhancing viral diffusion nearby from a potentially infected patient. The maneuver itself, which involves a maximum inspiration followed by an explosive and prolonged expiration, induces cough and the release of droplets in the environment (4). To note, young patients often require a high number of attempts to achieve a reliable flow-volume curve (13, 14).

## Measurements of Respiratory Resistance

Alternative tests are already available in many pulmonary function laboratories. The forced oscillation technique (FOT) provides information on airway patency/responsiveness in terms of respiratory system impedance (Z_rs_) and its components, respiratory resistance (R_rs_) and reactance (X_rs_), during the patient's quiet breathing. Small oscillations are applied passively with a wave-emitting source (a loudspeaker) connected to the patient's mouth opening while cheeks are supported by the child or an operator. The pressure and flow relationship are then used to calculate R_rs_, X_rs_, the resonant frequency (F_res_), and the area under the reactance curve (AX) (15). Waves can be delivered as a single frequency or multiple-single frequencies; another delivery method employs a train of pulses, the so-called impulse oscillometry (IOs) (15–17). Among the Z_rs_ components, X_rs_ represents counteracting inertial and elastic forces of the respiratory system (chest wall, lung, airway tissues, and gas column moving inside). Both R_rs_ and X_rs_ are frequency-dependent; thus, frequencies >4 HZ can easily reach the peripheral airways whereas high frequencies (>20 HZ) reflect frictional forces of the proximal airways and surrounding tissues (15, 16). Frequencies between 4 and 10 Hz are retained clinically relevant for children (16). For these frequencies, the within-subject coefficient of variation in R_rs_ (CV = SD/mean × 100) varies between 6.2 and 8.0% for children aged 3–16 years (14). Both R_rs_ and X_rs_ measures are repeatable over 2 weeks (18). Day-to-day R_rs_ variability is higher in asthmatic children than in healthy controls; this variability is associated with disease severity and symptom control (19).

The clinical utility of FOT measurements for assessing airway obstruction and bronchial response to bronchodilators has been recently summarized (15, 16, 20). Differences between techniques and patients' selection criteria lead to contrasting results when airway patency of healthy and wheezy children is compared at baseline; instead, most studies agree in the ability of FOT for assessing changes in the airway caliber (Table 1). The entity of Z_rs_ largely depends on standing height; thus, laboratories need to construct their reference values or to adopt those appropriated to their population (15). A decrease of at least −40% in R_rs_ and an increase of at least +50% in X_rs_ are considered thresholds for a bronchodilator response (15). Still, bronchodilator doses differ between studies (15, 20). Though several reference values have been provided for the different techniques, a call for standardization of these techniques is still evident (15–17, 20). The use of reference equations from studies whose population and devices most closely approximate the local situation has been recommended (15). In the absence of appropriated reference equations, the “personal best” measurement could be recorded as a reference point for the individual patient, to guide therapeutic decisions.

**Table 1:** Alternative pulmonary function tests (PFTs) for asthmatic children during the Covid-19 pandemic.

**Laboratory (References)**	**Advantages**	**Limitations**
FOT (9, 10, 14–21)	• Assess respiratory mechanics and airway resistance during tidal breathing. Help to detect peripheral airway obstruction. • Brief, feasible for children who are unable to cooperate with spirometry. • Baseline outcomes fairly distinguish subjects with recurrent wheeze/asthma from those healthy (see limitations). • Best utility, to assess the BDR and AHR. • Help overtime assessment and prediction of loss of asthma control.	• Outcomes depend on patient selection and diagnostic criteria. • Sensitive to upper airway shunting. • Multi-ethnic normative values are lacking. • Usefulness for long-term monitoring of patients, further studies needed. • Standardization of the technique and response to bronchodilators (type, drug dose, and timing), should be improved.
Rint (9, 14, 18, 22–25)	• Assess respiratory resistance during tidal breathing. Simple, quick, adapted for toddlers. • Reported high values in young children with persistent wheeze as compared with transient wheezers or never wheezers. • Assess the BDR with good sensitivity and specificity. • Relatively useful to assess AHR to cold air or exercise challenge (see limitations).	• Low sensitivity to detect peripheral airway obstruction. • Sensitive to upper airway shunting. • Does not discriminate well between children with recurrent wheeze and those healthy. • May underestimate resistance in children with severe airway obstruction. • Unclear utility for asthma monitoring.
FE_NO_ (26–33)	• Assess TH2-type airway inflammation during slow exhalation maneuvers. • Moderate accuracy for asthma diagnosis in subjects 5 yrs. and older. • Patients with FE_NO_ >35 ppb are likely to benefit from inhaled corticosteroids (ICs). • Assist correct use of ICs, therapy compliance, and resistance to ICs. • Help to monitor biological therapy. • Raising levels predict disease exacerbations.	• Positively skewed levels; overlapping between asthmatic and healthy subjects. Low FE_NO_ does not exclude asthma. • The optimization of therapy based on FE_NO_ has not proven better outcomes. • Several factors can affect its levels (e.g., atopy, infections, comorbidities, age, height, sex, and smoking exposure). • Needs coaching, especially in young children.
MBW (18, 34–40)	• Inert gas clearance technique. Assess ventilation distribution inhomogeneity during tidal breathing. Also measures the functional residual capacity (FRC). • Feasible for young children, reproducible. • Useful in severe or uncontrolled asthma. • More sensitive than spirometry to detect small airway disease. • Both MBW and FE_NO_ indices can help to assess disease exacerbations and EIB.	• Prolonged testing, especially in patients with uneven ventilation. • Requires experienced personnel. Preparation of the equipment and data processing is complex. • Insensitive to detect small airway dysfunction in mild asthma. • Multi-ethnic normative values are lacking. • Expensive devices, scarce accessibility.
**Home (References)**		
PEF (41–45)	• Assess airflow limitation during maximal expiratory maneuvers. Hand-held devices.	• Effort dependent. Do not enhance self-management during asthma flare-ups.
	• Assessment of diurnal variation or changes between visits; variability weakly correlates with asthma symptoms and AHR. • New electronic devices with smartphone applications are feasible for children.	• Written records are unreliable. • Compliance decreases after 4 weeks. • Often disagrees with spirometric records. • Electronic PEF meters with automatic teletransmission still need validation.
Spirometry (13, 46–50)	• Assess maximal inspiratory and expiratory volumes; estimate the baseline airway patency and its changes (BDR and AHR). • Flow-volume curves can be evaluated remotely, by an operator. • Acceptability and reproducibility criteria (with instructions to subjects if criterion not met) are available. • Portable devices.	• Effort dependent; underestimated data. Data quality decreases with younger age, lack of controller therapy, and FEV_1_ < 80%. • Daily FEV_1_ telemonitoring does not lead to better symptom control or fewer attacks. • Devices often lack instructive videos and maneuver's quality feedback. • Variable accuracy. Expensive. • Smartphone spirometers need validation.
FOT (51)	• As above (Laboratory). Useful for assessing day-to-day variability.	• Expensive. Requires more evidence for long-term monitoring.
FE_NO_ (52, 53)	• As above (Laboratory). Improves with mobile direct observation of therapy (MDOT).	• Expensive. Needs good quality control, instructions, and online feedback.

*FOT, forced oscillation technique; Rint, respiratory resistance measured with the interrupter technique; FE_NO_, fractional exhaled nitric oxide concentration; MBW, multiple breath washout; PEF, peak expiratory flow; BDR, bronchodilator response; AHR, airway hyperresponsiveness; ICs, inhaled corticosteroids*.

A modified FOT method that measures the tidal volume dependence on airway resistance has been recently described (21). This method, based on the change of within-breath R_rs_ at zero flow (end-expiration vs. end-inspiration), improves the ability to detect acute airway obstruction in young children (21). Advantages of FOT over spirometry and other tests of airway resistance have been described recently (9). Yet, a systematic review could not disclose enough evidence to place FOT as an adjunct or as a substituent of spirometry (10). Despite the FOT gaining interest, the lack of familiarity with the technique, lack of equipment, and complexity of analysis still limit its wider adoption.

Estimates of airway resistance can be also obtained with the interrupter technique (Rint). The child is invited to sit with the neck in a neutral position, to wear a nose clip, and to breathe normally through a mouthpiece and microbial filter. An automated valve briefly occludes the airway opening at the end-expiratory phase, lasting 100 ms; the resultant pressure is divided by the flow immediately preceding the interruption to estimate airway resistance (14). As for the oscillometer, cheek support is required. The assumption is that alveolar and mouth pressure equilibrate after occlusion. This technique is simple, quick to perform, and suitable in younger children. Rint measurements mostly represent airway resistance but also a small resistance from lung tissues and chest wall (14). The within-subject variability in healthy children is close to 12% (14, 18). However, the wide between-subject CV in health leads to overlap values with those obtained in children with recurrent wheeze (18). Baseline Rint measurements have low sensitivity to detect bronchial obstruction but perform better for assessing bronchial response to bronchodilators (BDR) (14, 22, 23). A reliable BDR has been reported for a decrease in Rint ≥ 0.26 kPa L^−1^s from baseline or −1.25 Z-scores (22).

Measurements of respiratory resistance have a long history (14, 54); still, they must gain a wider consensus among general practitioners and pulmonologists. While airway resistance could be measured with body plethysmography, it has inconvenience in the pandemic context such as the need for thorough disinfection of the box and execution difficulties for younger children. Instead, oscillometry and Rint techniques are available with smaller devices and are simple to perform in the Lab. Another advantage of these procedures is that the operator can be placed behind the child during the breathing maneuvers and his participation to support the child's cheeks is required only for toddlers. Acquisition time is also reduced for these tests. For instance, during oscillometry, the minimal acquisition time for children under 12 years of age is 16 s (15). Both FOT and Rint devices are small and supplied with appropriated in-line antimicrobial filters. Some instruments (e.g., Resmon Pro Full) store outcomes automatically in a pen drive, so the operator can evaluate the results safely, outside the laboratory. The advantages and limitations of FOT and Rint are reported in Table 1.

## Exhaled Nitric Oxide (NO)

The fractional concentration of exhaled nitric oxide (FE_NO_) has been studied in the last decades for assessing asthmatic patients, including children; guidelines on standardized methods are still valid (26). This free radical is a helpful non-invasive biomarker of the atopic-eosinophilic (Th2-type) airway inflammation (26, 27). A robust model with FE_NO_, together with blood eosinophil counts and other biomarkers of IL-13–driven gene expression (serum CCL17 and CCL26), has been recently developed; this model identifies Type 2-high asthma patients with positive and negative predictive values of 100 y and 87%, respectively (28).

The test requires a deep inspiration followed by a constant slow exhalation into the analyzer across an in-line microbial filter. A target expiratory flow rate of 50 ml/s, against an expiratory resistance between 5 and 20 cmH_2_O, is required to close the soft palate and to exclude contamination from nasal NO. Exhalations of at least 4 s can be sufficient to achieve a NO plateau. Schoolchildren usually can perform the classic maneuver; toddlers need audiovisual coaching and the use of dynamic flow restrictors to maintain a constant expiratory flow rate (26).

FE_NO_ measurements help in asthma diagnosis if taken together with the clinical history; FE_NO_ also assists in evaluating the therapeutic response to oral or inhaled corticosteroids and predicts disease exacerbations after treatment withdrawal (29). Because FE_NO_ is a phenotype-linked biomarker, it is suited for monitoring children on biological therapy (30). A recent systematic review and meta-analysis support the diagnostic accuracy of FE_NO_ testing in pediatric asthma (31). Overall, FE_NO_ levels over 35 ppb in children indicate eosinophilic airway inflammation and changes below or above 20% between visits are consistent with either response to anti-inflammatory therapy or with a need for adjusting therapy, respectively (29).

Peripheral and proximal airway contributions of exhaled NO can be calculated from exhalations at several flow rates, using the two-compartment model (55). Their employ has been reported useful to distinguish disease patterns in asthmatic patients (32). However, most studies agree on the role of partitioned-NO airway parameters to assess exercise-induced bronchoconstriction (EIB), a hint of poor disease control (34, 56, 57). Recently, we found that increased concentrations of both alveolar NO (CaNO) and urinary adenosine predicted EIB in atopic asthmatic children (33). Because exercise bronchial challenge is an AGP, surrogates of EIB such as partitioned-NO parameters could replace the traditional challenge at this time.

An advantage of FE_NO_ testing is the low-target expiratory flow rate that needs only slow exhalation maneuvers. FE_NO_ analyzers are small; also, handheld devices help for daily home monitoring (Table 1).

## Multiple Breath Washout (MBW)

Small airway dysfunction and ventilation heterogeneity are relevant in asthma (11, 18, 35–37). Uneven ventilation can be assessed through inert gas dilution during tidal breathing: washout of the resident nitrogen with 100% oxygen, or initial wash-in of an exogenous gas (e.g., sulfur hexafluoride, SF_6_), and washout thereafter. Initial and final tracer gas concentrations allow measuring the resting volume [i.e., the functional residual capacity (FRC)]. When the dilution process achieves 1/40th of the initial gas concentration, the “Lung clearance index” (LCI) is then calculated as the cumulative expired volume (CEV) during the procedure divided by the FRC:

LCI = CEV/FRC.

This index means how many “turnovers” are required to clear the subject's FRC and reflects the extent of its ventilation distribution inhomogeneity. Young children usually cooperate with the test; they need coaching to breath normally while seated, wearing a mouthpiece with in-line microbial filter. Gas leaks should be avoided during the process (11, 38).

There is a slight inverse relationship between LCI and age in schoolchildren. Reports on upper limits for normal LCI vary between 7.0 and 7.9 in subjects aged 6–18 years, depending on the technique (39). Children with severe asthma have elevated LCI values as compared to those with a mild-to-moderate disease or healthy controls. However, many patients with severe disease yield LCI values within the normal range (11, 36).

Presence of high LCI values in subjects whose FEV_1_ is normal suggests ongoing small airway dysfunction (37). A recent study shows that both LCI and FE_NO_ (but not FEV_1_) concordantly improved 4 weeks after a systemic steroid dose in children with severe therapy-resistant asthma (35).

Some devices can analyze the phase III of each tidal breath to estimate the conductive (Scond) and acinar (Sacin) contributions to inhomogeneity. Scond better correlates with LCI than Sacin (11, 37).

The clinical application of these indexes still needs to be established. See also in Table 1. Using the MBW technique during this pandemic requires caution. Tidal breathing PFTs generate small particles (≤0.5 μm) at the equipment inhalation port, even if at lower amounts than forced expiratory maneuvers (58). The main concerns with MBW testing are prolonged breathing (and room stay) and possible gas leaks. Requirements for testing high-risk patients (see Introduction) are advisable.

## Home PFT Measurements

Several home PFTs have been developed with the aim to enhance patients' self-management (41, 47). Their use helps to overcome the infection control issues, as compared with laboratory tests. Most of these PFTs regard PEF and spirometry (13, 41–47, 51–53). Unsupervised measurements tend to be lower at home than in the laboratory, suggesting the need for patients' coaching (59, 60). Telemonitoring with visual coaching and automatic feedback for outcomes is promising, but its clinical utility remains unclear (13, 49). Daily home FEV_1_ telemonitoring did not reduce exacerbations in children with severe asthma (49). A recent Cochrane review found no additional benefits of telemonitoring for asthma control or exacerbations, over usual asthma care (50). Devices differ widely on their inclusion of instructive videos, graphical descriptions, and immediate feedback on the quality of the breathing maneuver (47) (Table 1).

## Conclusion

The unpredictable duration of the Covid-19 pandemic imposes infection prevention and control measures in ambulatory settings with a pulmonary function. In keeping with these preventive actions, pulmonary function procedures offering quiet breathing or slow expiration are suitable for testing asthmatic children. Measurements of airway resistance (Oscillometry, Rint), and FE_NO_, can help respiratory physicians to manage their patients until spirometry and bronchoprovocation tests can be resumed. The MBW test relies on the need to evaluate ventilation inhomogeneity in children with severe disease. Standardization of these PFTs and improvement of home telemonitoring are priorities, given this and other infectious community threats.

## Author Contributions

MB and MV conceived and designed the text body. MB wrote the manuscript. ME, MM, and SM helped for searching the literature and reviewed critically the manuscript. All authors contributed to the article and approved the submitted version.

## Conflict of Interest

The authors declare that the research was conducted in the absence of any commercial or financial relationships that could be construed as a potential conflict of interest.
